# OA09 Foetal Complete Heart Block in Ro positive Sjögren’s Syndrome patients- do we need a new prevention paradigm?

**DOI:** 10.1093/rap/rkae117.009

**Published:** 2024-11-05

**Authors:** Rohit Baslas, Kuljeet Bhamra

**Affiliations:** Wexham Park Hospital, Frimley Health NHS Foundation Trust, Slough, United Kingdom; Wexham Park Hospital, Frimley Health NHS Foundation Trust, Slough, United Kingdom

## Abstract

**Introduction:**

Sjögren’s Syndrome (SS) remains under recognised and frequently under treated. It affects approximately 0.6% of adults in the UK, with a mean age of 50 years. It is more common in women and young adults and thus pregnancy is not uncommon in affected women resulting in an increased impact of the disease on pregnancies. There is an increased rate of pregnancy complications including spontaneous abortion, foetal loss, congenital heart block (CHB) and neonatal lupus.

We present a case series of three pregnant patients with foetal congenital heart block, with varying outcomes, highlighting gaps in current knowledge and medical practice.

**Case description:**

Case 1: A female, born in 1986, suffering with dry eyes/mouth, was diagnosed with SS in 2018. Ro and La antibodies were intermittently positive during disease flare- mainly rash secondary to hypergammaglobulinemia. Mother had SS. In October 2022, an echo during the first pregnancy, at gestational age of 20 weeks+4 days with expected date of delivery (EDD) in March 2023, showed CHB with atrial and ventricular rate of 147 and 57 bpm respectively and no hydrops. Hydroxychloroquine (HCQ) 400 mg od and Dexamethasone 4 mg od were started.

She underwent weekly echo. At the end of October 2022, ventricular rate dropped to 50 bpm. Salbutamol was started. She underwent a caesarean-section (CS), a month before EDD and baby received temporary pacemaker, 2 days post CS.

Case 2: A female, born in 1997, had a history of intermittent joint pain, no sicca symptoms and Ro/La antibodies were positive. Mother had two miscarriages. In 2018, G1P1, she had CS at 36 weeks. Baby died soon after delivery due to CHB. In her second pregnancy in 2019, she underwent CS at 33 weeks and delivered a healthy baby with no complications. During third pregnancy in 2020, foetus developed congenital heart block from 16 weeks onwards. She delivered baby, via CS, two months before EDD. Baby died four months later due to multiple organ failure. In 2022, she delivered a full-term heathy baby, via CS. Foetus had CHB at 20 weeks, but it disappeared within a week.

Case 3: A female, born in 1988, G2P1 with a healthy child, had an echo in Jan 2023 at 18 weeks of pregnancy. Echo showed CHB with pericardial effusion, atrial and ventricular rate of 103 and 47 bpm respectively. Dexamethasone and salbutamol were started. At 24 weeks, foetal hydrops was present with no heart sounds.

**Discussion:**

All three patients had high Ro/La antibodies: Ro > 240 units/ml and La > 320 units/ml (normal <7unils/ml for both).

First patient was diagnosed with SS privately and was referred to us after an incidental finding of CHB at 20 weeks. Perhaps, HCQ was not prescribed prior to pregnancy due to intermittent nature of her symptoms and rheumatologist might have not been aware of her pregnancy. On discovery of CHB at 20 weeks of gestation, tertiary centre team started HCQ, although patient was advised that it won’t reverse CHB. Dexamethasone was also started as CHB was assumed to be recent, but it was later replaced with Salbutamol in two weeks due to inefficacy.

Second patient was advised to start HCQ after the first pregnancy, but it didn’t materialise. She was referred to us after first pregnancy and her subsequent pregnancies were managed at tertiary centre.

Third patient was referred to rheumatology after foetal death. We found that she had two-year history of hair fall, arthralgia, dry eyes, and dry mouth. Ro and La antibodies were positive. She has been advised to start HCQ preconception for future pregnancies and been made aware that HCQ can prevent CHB in 50% of foetuses and pregnancy outcomes can be adverse, despite best possible evidence-based care. A detailed history at the time of early pregnancy or before could have led to early diagnosis and initiation of HCQ, which could have prevented foetal demise.

CHB is associated with anti-Ro and/or La antibodies in the maternal circulation and prevalence is higher in those with high-titre antibodies. Prevalence rate of CHB is 1.8% in Ro/La antibody positive pregnant females, but recurrence rate is 18-20%. HCQ 400 mg OD reduces recurrence by 50%. Evidence shows Dexamethasone is not beneficial, once CHB has developed.

**Key learning points:**

• As the maternal age increases, it is likely that more and more patients will have an overlap of SS and pregnancy, potentially increasing the incidence of CHB.

• Given that HCQ prevents CHB in up to 50% of Ro positive pregnant females, if given before 10 weeks of pregnancy and CHB is usually detected at 16 to 28 weeks, we need to identify women with Ro antibodies before conception or before 10 weeks of pregnancy. This is complicated by the presence of Ro/La antibodies in up to 3% of general population and evidence suggesting that Ro/La antibodies can be present several years prior to the development of symptoms. Thus, a negative history of symptoms alone will not detect patients who may have Ro antibodies complicating pregnancy. Cardiac pacing has its own risks adding cost and morbidity.

• As a bare minimum, it is important that all patients with established SS in childbearing age be advised about risks due to SS in pregnancy including CHB, need for pacemaker for the baby soon after pregnancy if baby develops CHB, role of HCQ in decreasing the risk of CHB occurrence and absence of any other effective treatment currently making role of HCQ even more paramount.

• Key questions are whether we should have screening of all pregnant patients in the UK for SS/lupus symptoms and should we do routine Ro/La antibody check in early pregnancy? A deep dive into economies of scale for Ro/La testing cost, management of false positives, and risks and benefits of HCQ may foster development of a preventive strategy until effective treatments are in place.

